# Glucose, lipids and gamma-glutamyl transferase measured before prostate cancer diagnosis and secondly diagnosed primary tumours: a prospective study in the Swedish AMORIS cohort

**DOI:** 10.1186/s12885-018-4111-5

**Published:** 2018-02-20

**Authors:** Cecilia Bosco, Hans Garmo, Niklas Hammar, Göran Walldius, Ingmar Jungner, Håkan Malmström, Lars Holmberg, Mieke Van Hemelrijck

**Affiliations:** 1 King’s College London, Translational Oncology & Urology Research (TOUR) Division of Cancer Studies King’s College London Research Oncology, 3rd Floor, Bermondsey Wing, Guy’s Hospital, London, SE1 9RT UK; 2 Regional Cancer Centre, Uppsala, Sweden; 3 Unit of Epidemiology, Institute of Environmental Medicine, Karolinska Institutet, Stockholm, Sweden; 4 Department of Cardiovascular Epidemiology, Institute of Environmental Medicine, Karolinska Institutet, Stockholm, Sweden; 5 Department of Clinical Epidemiology, Karolinska Institutet and CALAB Research, Stockholm, Sweden; 6 Medical Evidence & Observational Research, Global Medical Affairs, AstraZeneca, Mölndal, Sweden; 7 Department of Surgical Sciences, Uppsala University, Uppsala, Sweden; 8 Swedish Orphan Biovitrum, AB Stockholm, Sweden

**Keywords:** Prostate cancer, Second primary tumours, Triglycerides, Gamma-glutamyl transferase, Glucose, Total cholesterol

## Abstract

**Background:**

Improvements in detection and treatment of prostate cancer (PCa) translate into more men living with PCa, who are therefore potentially at risk of a secondly diagnosed primary tumour (SDPTs). Little is known about potential biochemical mechanisms linking PCa with the occurrence of SDPTs. The current study aims to investigate serum biomarkers of glucose and lipid metabolism and gamma-glutamyl transferase (GGT) measured prior to PCa diagnosis and their association with the occurrence of SDPTS.

**Methods:**

From the Swedish AMORIS cohort, we selected all men diagnosed with PCa between 1996 and 2011, with at least one of the five biomarkers of interest (glucose, fructosamine, triglycerides, total cholesterol (TC), GGT) measured on average 16 years before PCa diagnosis (*n* = 10,791). Multivariate Cox proportional hazards models were used to determine hazard ratios (HR) for risk of SDPTs (overall and subtypes) by levels of the five biomarkers. Effect modification of treatment was assessed.

**Results:**

811 SDPTS were diagnosed during a median follow-up time of 5 years. Elevated levels of triglycerides (HR: 1.37, 95%CI: 1.17–1.60), TC (HR: 1.22, 95%CI: 1.04–1.42) and GGT (HR: 1.32, 95%CI: 1.02–1.71) were associated with an increased risk of SDPTs. Risk of SDPTs subtypes varied by biomarkers.

**Conclusion:**

Elevated levels of biomarkers of lipid metabolism and GGT measured prior to PCa diagnosis were associated with an increased risk of SDPTs, suggesting a potential common biochemical background for development of PCa and SDPTs.

## Background

Due to advances in detection and treatment, prevalence of prostate cancer (PCa) is increasing [1]. These trends translate into more men living longer with PCa diagnosis and consequently being at risk of getting diagnosed with a second primary tumour. Travis et al. suggested grouping of second primary malignancies based on leading etiological causes: treatment-related, syndromic, and those due to shared etiologic factors [1]. Most studies to date have focused on primary cancer treatment related second primary malignancies [2, 3] and little is known about other causes of these tumours. However, it was recently suggested that radiotherapy is only responsible for < 10% of second primary malignancies, implying a larger role for lifestyle, genetic or biochemical related factors in their development [4]. There are several definitions for second primary malignancies, and although most definitions contemplate the fact that it cannot be a metastasis of a first primary tumour, there is an implicit notion that second primary malignancies must have started developing after the first primaries. This implicit notion may be accurate for those second malignancies in which the lag time between treatment of the first primary tumour or long-term exposure to certain factors has taken place before the occurrence of the second primary malignancy. However, for those tumours diagnosed within relatively short period of time after diagnosis of first primary malignancies these factors are less likely to play a role in the development of a second primary malignancy. Here, we refer to second primary tumours as those primary tumours (non-metastatic) of any organ/tissue diagnosed after diagnosis of PCa. “Second” does not refer to when the tumour started developing, it refers to when it was diagnosed in relation to PCa diagnosis. Therefore, to emphasize this concept, below we will refer to these tumours as “secondly diagnosed primary tumours” (SDPTs).

Abnormal metabolism in cancer cells has been well studied and continues to be of high interest as a potential target for drug development [5]. It is therefore of interest to investigate serum biomarkers measured before a first primary cancer as these could activate mechanisms leading to other cancer development or be a consequence of common alterations that will lead to a second cancer [6].

Studies based on the Swedish Apolipoprotein MOrtality RISk (AMORIS) have reported that the interplay between serum lipids, glucose and gamma-glutamyl transferase (GGT) increases the risk of prostate cancer (PCa) [7–9]. Since cancer development following exposure to risk factors may take several decades [10], we hypothesized that these elevated serum levels of glucose, total cholesterol, triglycerides, fructosamine and GGT measured before PCa diagnosis may also be associated with development of SDPTs – either because these biomarkers activate a shared carcinogenic mechanism or because they are the consequence of a common underlying alteration [6, 11, 12] . We therefore conducted a hypothesis-generating study to evaluate how serum biomarkers of common metabolisms are associated with development of PCa and subsequent other cancers.

## Methods

### Study population

The AMORIS study has been described in detail elsewhere [13–15]. This database includes 812,073 Swedish men and women with blood samples evaluated by the Central Automation Laboratory (CALAB) in Stockholm, Sweden, during the period 1985–1996. Individuals recruited were either healthy and having laboratory testing as part of a general check-up or outpatients referred for laboratory testing. None of the participants were inpatients at the time of sampling ([16, 17]. The CALAB database was linked to several Swedish national registries such as the Swedish National Cancer Register, the Hospital Discharge Register, the Cause of Death Register, the consecutive Swedish Censuses during 1970–1990, and the National Register of Emigration using the Swedish 10-digit personal identity number [18–20].

**Fig. 1:**
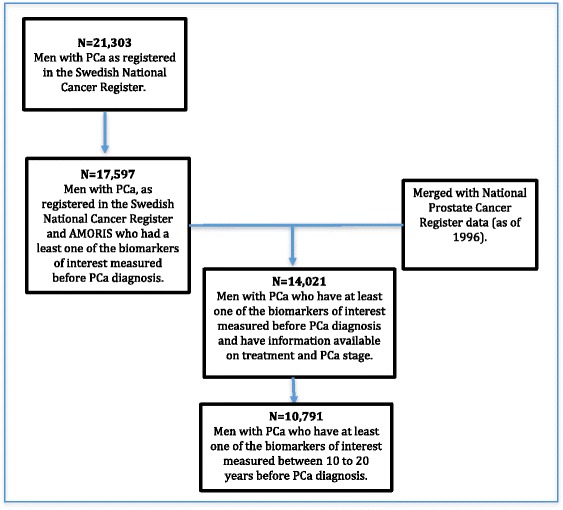
Selection of men with PCa from the AMORIS cohort to study the association between metabolism markers and risk of Second Primary Tumours

We selected all men diagnosed with PCa whom had at least one of the five biomarkers of interest (glucose, total cholesterol, triglycerides, fructosamine and GGT) measured before PCa diagnosis. To obtain information on stage and treatment of PCa, we used a linkage of the AMORIS cohort with the National Prostate Cancer Register (NPCR), resulting in a total of 14,021 PCa men [21]. As carcinogenesis may initiate several years before diagnosis, we divided the time between blood measurement and PCa diagnosis into 5 periods to exhibit this potential lag time: 0–5, 5–10, 10–15, 15–20 years and more than 20 years before PCa diagnosis. For the current study, we focused on those measurements taken 10–15 and 15–20 years prior to PCa diagnosis (*n* = 10,794 cases). Excluding the most recent periods limits the potential effects of reverse causation. Moreover, PCa is known to have a long natural history [22]. If men had more than one measurement taken within the period studied, the measurement closed to the mid-point of the interval was selected. Follow-up started at time of PCa diagnosis and ended at time of occurrence of a SDPT, emigration, death or end of the study (December 31st 2011), whichever came first (Fig. 1).

Information on educational level was retrieved from the Population and Housing Census for 1970–1990. Using information from the National Patient Register, we calculated the Charlson comorbidity index (CCI), which includes 19 diseases, with each disease category assigned a weight. The sum of an individual’s weights was used to create a score, resulting in four comorbidity levels ranging from no comorbidity to severe comorbidity (0, 1, 2, and ≥3) [23].

### Exposure variables

The main exposure variables of interest were the above-mentioned five biomarkers. We used both quartiles and medical cut-offs for the analyses. Medical cut-offs for glucose (6.11 mmol/L), total cholesterol (6.5 mmol/L) and triglycerides (1.71 mmol/L) were based on the National Cholesterol Education Program guidelines [24]. Fructosamine and GGT have less consistently established clinical cut-off levels, therefore these were defined based on the laboratory cut-offs used by CALAB which have also been applied in other recent studies (2.5 mmol/L and 36 IU/L respectively) [8, 25].

Although these biomarkers measurements were part of regular health check-ups and hence missing data can be considered to be missing at random, we used the most general approach to the problem of missing data: multiple imputation. Before imputing the data we performed a complete case analysis and observed associations were even stronger. The imputed data analysis results therefore reflect the most likely strength of the association by allowing all cases to be included and contributing to the analysis. More specifically, we applied multivariate imputation using chained equations (MICE), also known as imputation using fully conditional specifications [26]. The MICE method imputes multiple variables sequentially using univariate fully conditional specifications.

Glucose was measured enzymatically with a glucoseoxidase/peroxidase method. Total cholesterol was determined with the cholesterol oxidase/peroxidase (CHOD-PAP) assay and triglycerides with the glycerol phosphate oxidase/peroxidase (GPO-PAP) assay [18, 19]. GGT levels measurement was performed with an enzymatic colorimetric test using L-c-glutamyl-3-carboxy-4-nitroanilide as donor substrate at a temperature of 37 °C, which is the reference method recommended by the International Federation of Clinical Chemistry and Laboratory Medicine (IFCC) [27]. Fructosamine was measured by the Nitroblue Teterazolium (NBT) colorimetric technique based on the reducing ability of fructosamine in an alkaline solution [28].

All methods were fully automated with automatic calibration and performed at one accredited laboratory [18].

### Outcome definition

The main outcome of interest was the occurrence of cancer diagnosed after PCa diagnosis. SDPTs were defined as any non-benign and non-metastatic tumour, and grouped according to the International Classification of Diseases 7th revision (ICD7) codes [29] which are the codes used to enter the data in the register. Based on previous evidence [30], rectal SDPTs were grouped together with genitourinary tumours for anatomic reasons to account for possible effects of radiotherapy and health seeker bias (as with urologic cancers) in PCa.

### Statistical analysis

Baseline cohort characteristics were compared using descriptive statistics (Student’s t-test, Wilcoxon Rank Sum test, and Chi squared test). Multivariate Cox proportional hazards regression analysis with age as a timescale was used to determine hazard ratios (HR) and 95% confidence intervals (95% CI) of risk of SDPTs. We adjusted for the remaining biomarkers, fasting status, PCa treatment, CCI at PCa diagnosis, diabetes mellitus at PCa diagnosis, time between blood test and PCa diagnosis and education. Even though information on grade and treatment were both available, we only adjusted for cancer treatment to avoid collinearity – treatment is a well-accepted indication of disease severity. Furthermore, we ran an analysis according to the type of SDPT for those cancer groups that had at least 60 events leaving out other types of cancers (i.e. haematological). The assumption of proportionality of the Cox model covariates was tested by plotting Schoenfeld residuals on several of the imputed datasets [31]. To address the potential effects of PCa treatment we performed both a stratified analysis as well as an additional adjustment for PCa treatment.

The study complied with the Declaration of Helsinki and was approved by the Ethics Review Board of the Karolinska Institute.

Statistical Analysis Software (SAS) release 9.4 (SAS Institute, Cary, NC) was used for data management; Stata Statistical Software: Release 14 (College Station, TX: StataCorp LP) was used for imputation and data analysis.

## Results

Study population baseline characteristics for PCa cases with and without a SDPTs are described in Table 1. A total of 811 SDPTs (7.5%) were diagnosed during a mean follow-up time of 4.98 (3.36 SD) years.

**Table 1:** Baseline characteristics of study population based on imputation dataset 1

	SDPTs *n* = 811	%	No-SDPTs *n* = 9983	%
Age				
Mean (SD)	69.83 (7.07)		68.06 (7.89)	
< 63	133	16.40	2693	26.98
63–67.99	191	23.55	2417	24.21
68–73.99	253	31.20	2542	25.46
> 74	234	28.85	2331	23.35
Education				
High	226	27.87	3132	31.37
Middle	352	43.40	4088	40.95
Low/no	222	27.37	2619	26.23
Missing data	11	1.36	144	1.44
Glucose (mmol/L)				
Mean (SD)	5.10 (1.28)		5.09 (1.19)	
> 6.11	54	6.66	661	6.62
< 6.11	724	89.27	8672	86.87
Missing data	33	4.07	650	6.51
Triglycerides (mmol/L)				
Mean (SD)	1.64 (1.26)		1.53 (1.06)	
> 1.71	270	33.29	2751	27.56
< 1.71	530	65.35	7028	70.40
Missing data	11	1.36	204	2.04
Cholesterol (mmol/L)				
Mean (SD)	6 (1.02)		5.91 (1.05)	
> 6.5	275	33.91	2797	28.02
< 6.5	526	64.86	7016	70.28
Missing data	10	1.23	170	1.70
Fructosamine (mmol/L)				
Mean (SD)	2.11 (0.32)		2.11 (0.26)	
> 2.5	31	3.82	296	2.97
< 2.5	575	70.90	7307	73.19
Missing data	205	25.28	2380	23.84
GGT (IU/L)				
Mean (SD)	36.98 (97.36)		32.30 (35.29)	
> 36	69	8.51	701	7.02
< 36	688	84.83	8424	84.38
Missing data	54	6.66	858	8.59
CCI at Pca dx				
0	606	74.72	7705	77.18
1	131	16.15	1451	14.53
2	45	5.55	466	4.67
3+	29	3.58	361	3.62
Diabetes at PCdx				
Yes	42	5.18	418	4.19
No	769	94.82	9565	95.81
FUT	3.77 (2.83)		5.28 (3.22)	
Treatment				
RT	101	12.45	1040	10.42
RP	175	21.58	2936	29.41
HT	206	25.40	2211	22.15
DT (AS WW)	231	28.48	2332	23.36
Unspecified	55	6.78	823	8.24
Missing data	43	5.30	641	6.42
Stage group				
Low risk	205	25.28	3140	31.45
Intermediate risk	251	30.95	2791	27.96
High risk	225	27.74	2220	22.24
Regionally metastatic	41	5.06	567	5.68
Distant metastases	59	7.27	928	9.30
Missing data	30	3.70	336	3.37

Abbreviations: *GGT* gamma glutamyl transferase; *PCa* prostate cancer; *SDPTs* secondly diagnosed primary tumours; *CCI* Charlson comorbidity index; *FUT* follow up time; *RT* Radiotherapy; *RP* radical prostatectomy; *HT* hormonal treatment, DT: deferred treatment

Multivariate analysis including all biomarkers studied showed a higher risk of SDPTs for those with high serum levels (above established clinical cut-offs) of triglycerides (HR: 1.37, 95%CI: 1.17–1.60), total cholesterol (HR: 1.22, 95%CI: 1.04–1.42) and GGT (HR: 1.32, 95%CI: 1.02–1.71), as compared to the normal levels (Table 2). When looking at quartiles only those in the 4th quartile of triglycerides were at higher risk of SDPTs, as compared to the first quartile. A weaker positive association with risk of developing a SDPT was also observed for those in the 4th quartiles of total cholesterol and GGT, as compared to the first quartile.

**Table 2:** Univariate and multivariate Cox proportional hazards regression analysis for risk of SDPTs by levels of serum biomarkers (Glucose, triglycerides, total cholesterol, GGT, and fructosamin)

		Univariate		Multivariate**		*p*-value/trend
	Variables	HR	95%CI	HR	95%CI	
Clinical cut-offs	Glucose (6.11 mmol/L)	1.11	0.84–1.46	0.87	0.64–1.19	0.42
	TG (1.71 mmol/L)	1.37	1.18–1.58	1.37	1.17–1.6	< 0.001
	TC (6.5 mmol/L)	1.33	1.15–1.54	1.22	1.04–1.42	0.01
	GGT (36 IU/L)	1.37	1.07–1.76	1.32	1.02–1.71	0.029
	FAMN (2.5 mmol/L)	1.27	0.88–1.82	0.91	0.59–1.39	0.64
Quartiles	Gluc-q1 (< 4.6 mmol/L)	1	ref	1	ref	0.56
	Gluc-q2 (4.6-5 mmol/L)	1.29	1.06–1.57	1.25	1.03–1.52	
	Gluc-q3 (5–5.4 mmol/L)	1.09	0.89–1.33	1.01	0.83–1.24	
	Gluc-q4 (> 5.4 mmol/L)	1.15	0.95–1.39	0.98	0.8–1.19	
	TC-q1 (< 5.2 mmol/L)	1	ref	1	ref	0.09
	TC-q2 (5.2–5.8 mmol/L)	1.1	0.88–1.36	1.05	0.84–1.3	
	TC-q3 (5.8–6.6 mmol/L)	1.06	0.87–1.3	0.98	0.8–1.49	
	TC-q4 (> 6.6 mmol/L)	1.38	1.14–1.67	1.21	0.99–1.49	
	Tg-q1 (< 0.9 mmol/L)	1	ref	1	ref	0.004
	Tg-q2 (0.9–1.3 mmol/L)	0.95	0.76–1.18	0.94	0.75–1.17	
	Tg-q3 (1.3–1.9 mmol/L)	1.11	0.91–1.34	1.08	0.88–1.31	
	Tg-q4 (> 1.9 mmol/L)	1.35	1.12–1.62	1.32	1.08–1.61	
	Ggt-q1 (< 16.79 IU/L)	1	ref	1	ref	0.27
	Ggt-q2 (16.79–23.39 IU/L)	1.07	0.87–1.32	1.05	0.86–1.29	
	Ggt-q3 (23.39–35.99 IU/L)	1.05	0.86–1.29	0.99	0.8–1.22	
	Ggt-q4 (> 35.99)	1.24	1.01–1.52	1.15	0.93–1.42	
	Famn-q1 (< 2 mmol/L)	1	ref	1	ref	0.26
	Famn-q2 (2–2.11 mmol/L)	0.88	0.69–1.11	0.84	0.66–1.07	
	Famn-q3 (2.11–2.25 mmol/L)	0.97	0.79–1.21	0.91	0.73–1.14	
	Famn-q4 (> 2.25 mmol/L)	0.99	0.8–1.24	0.85	0.67–1.07	

Abbreviations: *Gluc* glucose; *TC* total cholesterol; *TG* triglycerides; *GGT* gamma glutamyl transferase; *FAMN* fructosamine; *PCa* prostate cancer; *SDPTs* secondly diagnosed primary tumours; *HR* Hazard ratios; *CI* Confidence intervals

^**^Adjusted for: Education, diabetes mellitus at PCa diagnosis, age, CCI, Fasting Status, time between date of blood test and PCa diagnosis date, PCa treatment: Hormonal, Radiotherapy, Radical prostatectomy

Cancer treatment stratified analysis did not substantially change these associations (results not shown).

**Fig. 2:**
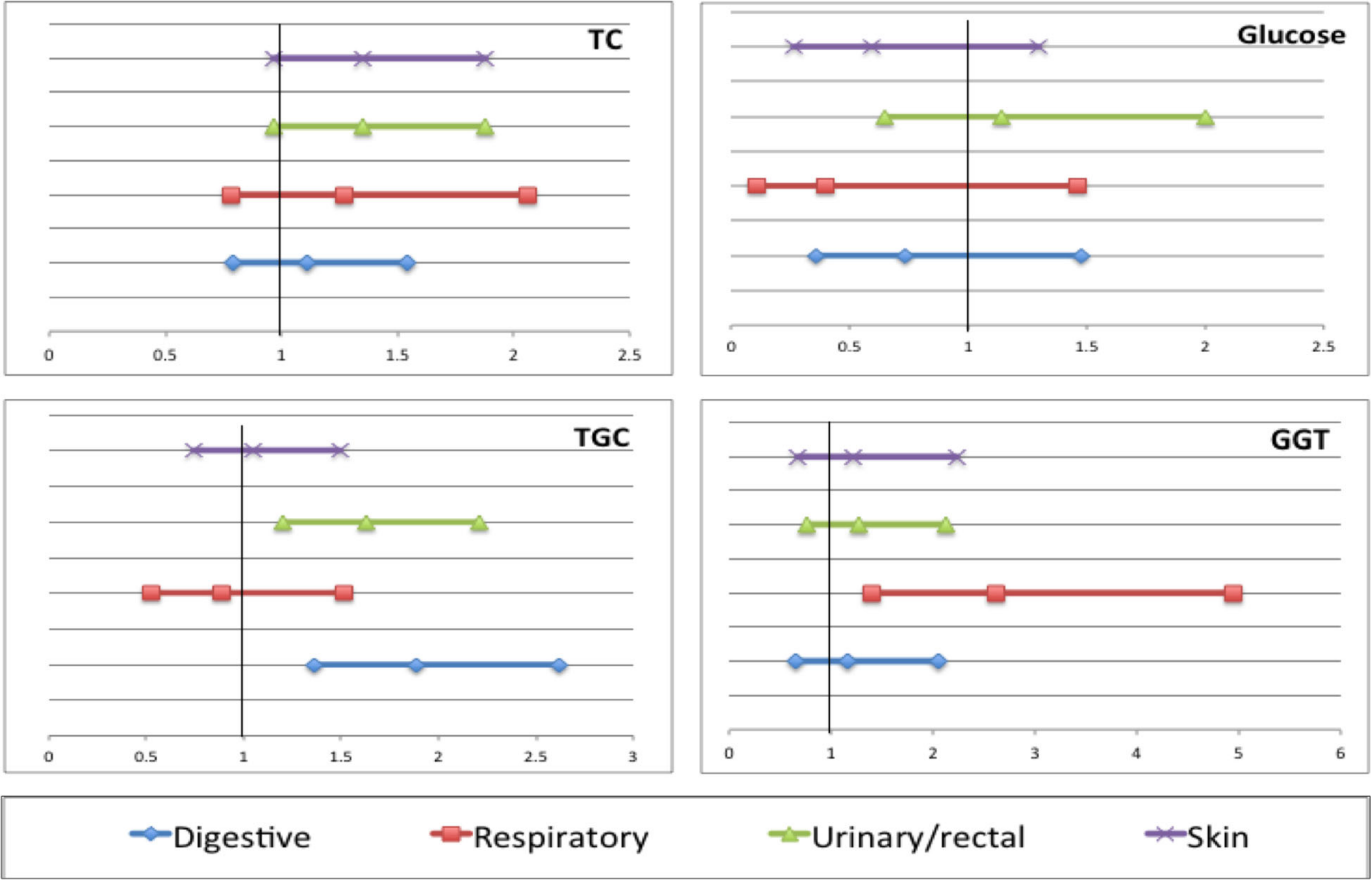
Hazard ratios and 95%CI (X-axis) for risk of specific types of SDPTS by levels of total cholesterol (TC), glucose, triglycerides (TGC), and GGT based on their medical cut-off (6.5 mmol/L, 6.11 mmol/L, 1.71 mmol/L and 36 IU/L respectively). All models were adjusted for education, diabetes mellitus at PCa diagnosis, age, CCI, Fasting status, time between date of blood test and PCa diagnosis date, PCa treatment: Hormonal, Radiotherapy, Radical prostatectomy

The risk of SDPTs of digestive organs, peritoneum, genitourinary and rectum was also higher for those with elevated blood levels of triglycerides. Furthermore, high levels of GGT were associated with SDPTs of the respiratory system. Total cholesterol levels were borderline significant for the risk of skin and genitourinary and rectum SDPTs (Fig. 2).

## Discussion

To our knowledge this is the first study investigating an association between serum markers of lipids, glucose and GGT and development of SDPTs in men with PCa. High levels of cholesterol, triglycerides and GGT measured on average 16 years prior to PCa diagnosis were associated with an increased risk of developing a SDPT. When looking at specific types of SDPTs we found an increased risk of SDPTs of digestive organs, peritoneum, genitourinary and rectum for those with elevated levels of triglycerides. High levels of GGT were also associated with an increased risk of SDPTs of the respiratory system.

Carcinogenesis is a complex process that requires several components to act/occur, at different points in time, before irreversible disease develops [32]. Our findings for SDPTs were consistent with our and other previously published findings for these biomarkers and primary PCa [8, 11, 33, 34]. Interestingly, results from a recent nested case-control study on the association between circulating fatty acids and PCa showed that those who had been diagnosed 10 or more years after blood collection had stronger associations than those diagnosed less than 10 years after blood collection. These findings support the hypothesis that metabolic factors may play a role several years before disease occurrence and detection [35].

Below we describe our findings in the context of other epidemiological studies as well as the hallmarks of cancer [10, 36].

### Lipid metabolism

Elevated levels of cholesterol and triglycerides are associated with a higher risk of PCa, gastrointestinal and renal cancer [37, 38]. In the AMORIS study these associations varied by levels of glucose [14, 34, 39]. Our findings for risk of SDPTs corroborate these observations, suggesting that PCa, gastrointestinal and genitourinary cancers may share a common lipid metabolism phenotype. In the context of melanoma, few epidemiological studies have investigated links with lipid metabolic alterations [40].

Cholesterol is necessary to build cell membrane and preclinical studies suggest that low levels of cholesterol cause cell cycle arrest. Furthermore, high levels of cholesterol induce a chronic inflammatory state, and thus potentially cell proliferation [41]. Recent experimental data also suggest that statins, a commonly used cholesterol-lowering drug, may impair cell proliferation and induce apoptosis [42–44].

### GGT

Our results show that elevated levels of GGT measured before PCa diagnosis are associated with higher overall SDPTs risk and more specifically with SDPTS of digestive organs and lung cancer. Epidemiological studies have established an association between GGT and several primary cancers. For instance, elevated levels of GGT are associated with increased risk of cancer in men [45], specifically PCa [8].

Several mechanisms have been suggested explaining the role of GGT in cancer cell proliferation and survival. Some of these processes include the recovery of essential aminoacids like glutamic acid and cysteine and balancing the reactive oxygen species (ROS) levels [46].

### Glucose and fructosamine

In contrast to previous findings in the AMORIS study [9], our results did not support a link between markers of glucose metabolism and development of SDPTs in men with PCa. This discrepancy in findings could be due to glucose measurements being potentially more sensitive to the time window before carcinogenesis. Our previous study focused on primary tumours and hence the time window between glucose measurement and risk of cancer was shorter than in the current study [9].

### Strengths and limitations

Studying the common aetiology of SDPTs is broad and difficult to implement. Using AMORIS, and its linkages to well-documented registries, allowed us to establish a stable association between the biomarkers of interest and development of SDPTS – which informs future hypotheses for understanding the process of carcinogenesis. Further strengths of the present study are the large number of men with prospective measurements of biomarkers measured at the same clinical laboratory with a clearly documented methodology. Missing data for the biomarkers studied was limited and multiple imputation was used to address this. Not enough data was available to perform longitudinal analysis including repeated biomarker measurements, a study design that would benefit future studies aiming to evaluate the effect of serum biomarkers on development of PCa and subsequent cancers. Use of national health registers provided complete follow-up for each person as well as detailed information on cancer diagnosis, time of death and emigration [47]. Subjects included in the study were mainly healthy at baseline as these measurements were to a large extent part of routine health check-ups in the occupational setting. The selection of biomarkers followed findings in previous AMORIS studies [7–9], but could be widened in future studies, though one then has to be careful for multiple comparisons.

PCa treatment is unlikely to impact on our study as a confounder. Although cancer treatment potentially increases risk of SDPTs, the induction period is usually > 5 years [48, 49]. Our follow-up time was on average five years, suggesting that SDPTs captured were probably already being developed by the time of PCa diagnosis. Moreover, it is important to note that those men diagnosed with an advanced PCa might be less likely to develop a SDPT due PCa-specific death being a competing risk. Given that this study was set up to generate hypotheses in the aetiological setting, we aimed to establish potential associations between biomarkers for development of PCa and other SDPT whilst taking into account PCa characteristics at time of diagnosis by adjusting for PCa-specific treatments. We therefore believe that competing risk are not strongly affecting the potential aetiological connections observed here.

Limited data on lifestyle factors was available, however all our models were adjusted for CCI, which indirectly accounts for effects of lifestyle (e.g., smoking-related diseases). Another limitation is the lack of information on drug prescriptions related to the biomarkers studied (e.g. anti-diabetes drugs). The Prescribed Drug Register only starts recording in Sweden in July 2015 [50]. Adjustment for CCI can therefore be considered as a crude proxy for the potential use of these drugs. Detection bias (i.e. those with higher levels of the biomarkers were more closely followed up by their physicians and therefore secondary tumours would have been detected more promptly) is not affecting this study, as we did not focus on biomarkers measured after PCa diagnosis. Detection bias of urological cancers is plausible due to closer specialist follow-up, however again this does not discard a possible biological link.

## Conclusion

Biomarkers of lipid metabolism as well as GGT measured before PCa diagnosis were associated with a higher risk of developing a SDPT. We have previously linked lipids and GGT [7, 8] with risk of PCa and can therefore suggest that SDPTs and PCa could share common biological components, involving the metabolism or potential effect of lipids or GGT – a hypothesis which will require future pre-clinical as well as longitudinal observational studies to corroborate.

## Abbreviations

95%CI: 95% Confidence Interval
AMORIS: Apolipoprotein MOrtality RISk
CALAB: Central Automation Laboratory
CCI: Charlson Comorbidity Index
CHOD-PAP: Cholesterol oxidase/peroxidase
GGT: Gamma glutamyl transferase
GPO-PAP: Glycerol phosphate oxidase/peroxidase
HR: Hazard Ratio
ICD: International Classification of Diseases
IFCC: International Federation of Clinical Chemistry and Laboratory Medicine
MICE: Multivariate imputation using chained equations
NBT: Nitroblue Teterazolium
NPCR: National Prostate Cancer Register
PCa: Prostate Cancer
ROS: Reactive oxygen species
SDPT: Secondly Diagnosed Primary Tumour
